# Development of a rapid and fruit-saving method for fatty acid composition analysis in olive: a comparative study on 27 cultivars

**DOI:** 10.3389/fpls.2024.1457518

**Published:** 2024-09-04

**Authors:** Marianna Rizzo, Gianluca Godino, Enzo Perri, Samanta Zelasco, Luca Lombardo

**Affiliations:** Council for Agricultural Research and Economics (CREA) Research Centre for Olive, Fruit and Citrus Crops, Rende, Italy

**Keywords:** fatty acids, *Olea europaea* L., olive oil, olive paste, oleic acid trend, new method

## Abstract

Fatty acid composition is an essential aspect of the qualitative assessment of olive oil. A method for evaluating and trending fatty acid composition of olive varieties directly from a limited amount of drupes, has been proven reliable in comparison with traditional oil analysis. No significant difference was detected between the two methods for the 27 cultivars tested, despite presenting decidedly different acid compositions. The results obtained, crossed with those of oil yield, can represent a useful resource to set the harvest calendars by choosing the most suitable time for the production of superior quality oils and for reducing the risk of pathogen infections or pest attack. For three cultivars, the acid composition was evaluated during three ripening phases (green, veraisoned and veraisoned to black). The different behaviors suggest it is the genotype that determines this -still little known- physiological trait in olive. An interesting finding was that the oils from drupes harvested in August showed linolenic acid values higher than the limit (1.00%) set in the international standards for the classification of olive oils, requesting further investigation.

## 1 Introduction

The olive tree (*Olea europaea* L.) is an identitarian species of the Mediterranean Basin, accounting for more than 90% of the world’s olive oil production and about 60% of its consumption (Hatzopoulos et al., 2002; D’Angeli and Altamura, 2016; IOC, 2021). Accordingly, due to its acclared nutritional and functional properties (Aguilera et al, 2005), olive oil consumption is steadily increasing all over the world even in non-producing countries (Salas et al., 2000; Mannina et al., 2001; Matteucci et al., 2011). An important role in the health properties of olive oil can be attributed to its peculiar composition in triacylglycerols (TAGs) -esters of glycerol and fatty acids (FAs)-, constituting the so-called saponifiable fraction (De Carvalho and Caramujo, 2018), for the most part made up of monounsaturated fatty acids (MUFAs) and in particular oleic acid (Lombardo et al., 2018). The importance of the quality of FAs in the diet is carved in the qualified health claim for olive oil by the Food and Drug Administration reporting that: “Limited and not conclusive scientific evidence suggests that eating about 2 tablespoons (23 grams) of olive oil daily may reduce the risk of coronary heart disease due to the monounsaturated fat in olive oil. To achieve this possible benefit, olive oil is to replace a similar amount of saturated fat and not increase the total number of calories you eat in a day.” In support of this, several studies have certified how the lipid composition of extra virgin olive oils (EVOOs) induces a broad spectrum of antiatherogenic responses, by reducing the plasma cholesterol contained in the low-density and very low-density lipoproteins (LDLs and VLDLs; Lu et al., 2024), by protecting LDLs from oxidation (Covas et al., 2015), by reducing platelet aggregation (Lopez-Miranda et al., 2007), and by modulating the expression of proatherogenic genes (Llorente-Cortés et al., 2010), resulting in a notable decrease of cardiovascular mortality (Schwingshackl and Hoffmann, 2014).

The content of each FA in olive oil is regulated by the legislation of the European Union (EU, 2022) and by the trade standard applying to olive oils and olive pomace oils set by the International Olive Council (IOC, 2019), as the FA composition of an oil is correlated to its quality and authenticity (Wabaidur et al., 2016). In fact, FA analysis can be used to detect commercial frauds, such as the adulteration of high quality olive oil with different types of oil (Christy and Egeberg, 2006). Further, knowing the FA composition of an oil is important to evaluate its degradation and to optimize the oil refining (Díaz and Borges, 2012). Consequently, equally important is the possibility of recording the acid profile trend/changes (if any) throughout the ripening of the drupes, so as to plan the best harvest time.

Commonly, FA composition of a lipid matrix is determined by gas chromatographic (GC) analysis (Greco et al, 2022) after extraction of the oil and methylation of FAs. In addition, liquid chromatography coupled with various detection techniques, including ultraviolet-visible adsorption, fluorescence and photo diode array, has been reported in literature for the analysis of FAs, after pre- or post-column derivatization with appropriate chromophores (Lima and Abdalla, 2002). Mass spectrometer was used as detector by some authors as well (Wabaidur et al., 2016).

The aim of the study was the development of a method to analyze FAs composition of olive varieties directly from a limited sample of drupes (ca 10 g) regardless of the ripening stage. This procedure allows trending FAs even when the oil yield is low, i.e. at early stages of the oil biosynthesis in olive drupes, during the “off “production years, or when the production of oil is not necessary, i.e. in the case of table olives. The analysis were carried out in parallel with the analysis of FAs in oils produced using an olive mill, in order to evaluate the goodness of the method.

## 2 Materials and methods

Fruit samples were collected from the Olive World Germplasm bank (OWGB) of the Italian Research Centre for Olive, Fruit and Citrus Crops (CREA-OFA). The international olive germplasm collection is located in Mirto Crosia, on the Ionian side of Northern Calabria (39° 36’ 54.1’’ North latitude, 16° 46’ 11.0’’ East longitude, 6 m a.s.l.) and, to date, collects 458 cultivars for a total of ca. 3000 olive trees, spaced with a regular planting pattern of 6x4 m in about 10 ha. Olive fruits were sampled by handpicking from a single tree per each cultivar between August and December 2023. Ripening stage was evaluated through the Jaén index (International Olive Council, 2011). A total of 10-20 kg of drupes per each of the 27 tested cultivars were collected and employed for olive oil extraction and determination of fatty acid composition from both oil and olive paste, and for oil yield. For three cultivars (Nocellara del Belice, Coratina and Tonda di Cagliari), acid composition and oil yield were evaluated during three ripening phases (I: in August on green drupes, II: in October/November on veraisoned -purple- drupes and III: in December on veraisoned to black drupes) repeating drupe sampling from the same trees.

The most part of the harvested fruits was milled for olive oil production within 24 hours. The remaining part, around 100 g of olives destined to be crushed for oil yield and determination of FA composition, was stored at -20°C until analysis.

### 2.1 FA analysis

FA composition of each cultivar from oil/olive paste was evaluated by oil extraction through mill/volatile solvents, and fatty acid methylation, followed by gas chromatographic analysis.

Chemicals and solvents used for the extraction were ACS gradient or equivalent. 2N methanolic KOH was prepared dissolving 11.2 g of potassium hydroxide (Merck KGaA, Darmstadt, Germany) in 100 ml of methanol (Honeywell, Seelze, Germany) containing not more than 0.5% (m/m) water. For GC analysis, *n*-hexane (Honeywell, Seelze, Germany) was chromatography quality.

#### 2.1.1 FA analysis from olive oil

Ten up to 20 kg of olives for each sample were milled in a *Oliomio Stile* mill (Mori-TEM, Barberino Tavarnelle, Florence, Italy) equipped with a knife crusher, a vertical continuous malaxer and a 2 phase decanter. The olive meal obtained was malaxed for 20 min at room temperature and the oil separated by centrifugation. Samples were stored in amber glass bottles.

FA methylation was carried out following the procedures described in the IOC guidelines (2017) with minor modifications. Briefly, 0.15 g of oil were added with 0.1 ml of a methanolic solution of KOH (2N) and dissolved in 1 ml of *n*-hexane. The resulting solution was shaken vigorously for 5 min and rested until stratification, with the fatty acid methyl esters (FAMEs) recovered in the upper layer. 0.25 ml of supernatant were transferred in a GC vial and added with *n*-hexane to the volume of 1.5 ml. The mean of the data was calculated from three biological repeats.

#### 2.1.2 FA analysis from olive paste

Olive samples stored at -20°C were left at room temperature overnight. Subsequently, they were milled using a stainless steel hammer crusher so as to obtain an olive paste. Ten grams of paste were weighted in a stoppered boiling tube and added with 25 ml of *n*-hexane, vigorously mixed for 30 seconds and left to extract overnight. Five ml of the clear supernatant from the olive meal extract were transferred to a clean boiling tube, added with 0.5 ml of methanolic KOH (2N) and mixed for approximately 30 seconds using a vortex-mixer. The solution was allowed to settle until a clear supernatant layer was obtained, before transferring some of the supernatant to a GC vial to proceed with GC analysis. The mean of the data was calculated from three biological repeats.

#### 2.1.3 Gas chromatographic analysis

FAMEs were separated and quantified using an Agilent 6890N GC instrument (Agilent Technologies, Santa Clara, CA, USA) equipped with a split injector 7683 series (Agilent Technologies, Santa Clara, CA, USA) and a flame ionization detector (FID). A highly polar capillary column SP-2340 (length = 60 m, i.d. = 0.25 mm, film thickness = 0.20 μm) (Supelco), coated with a cyanopropyl polysiloxane stationary phase, was used to separate the FAMEs. Helium was used as carrier gas at a constant flow of 1.2 ml/min and 1:50 split ratio, and the injection volume was 1 μl. FID was maintained at 250°C. The initial oven temperature was 110°C held for 5 min, followed by a rate increase of 3°C min^-1^ up to 180°C and held for 15 min, increased of 4°C min^-1^ to 220°C and held for 2 min.

FAMEs were identified through a comparison of their retention times versus pure standards analyzed under the same conditions. They were quantified according to their area, obtained by integration of the peaks. Data were collected and processed using the Agilent ChemStation Software (Agilent Technologies, Santa Clara, CA, USA). The results were expressed as percentage of individual fatty acids in the lipid fraction.

### 2.2 Oil yield

Oil yield was determined from the previously obtained olive pastes by Fourier transform near-infrared (FT-NIR) spectroscopy. Spectral measurements of the olive paste were performed using a Bruker multi-purpose analyzer (MPA) FT-NIR spectrometer (Bruker Optik, GmbH, Ettlingen Germany) equipped with InGaAs detector and a 20-watt high intensity tungsten-halogen NIR light source. Instrument control and spectra analysis were performed using the OPUS spectroscopy software (Bruker Optik, GmbH, Ettlingen, Germany).

### 2.3 Statistical analysis

Data were subjected to uni- and multivariate analysis of variance (ANOVA and MANOVA), after verifying the requirements for the application. The HSD Tukey’s *post hoc* test was performed to define the significance of differences between means at 95% confidence level, using Past (v. 4.10) software (Hammer et al., 2001).

## 3 Results and discussion

### 3.1 Preliminary analyses

Sampling date, Jaén index values and oil yield percentage of the drupes used for FA profiling from the 27 considered olive cultivars are reported in **Table 1**. Olives were harvested between August and December 2023 and showed a Jaén index in the range of 0 for the drupes of the cv Bidh el Hammam, Grossa di Spagna, Coratina, Tonda di Cagliari, Arbequina, Nocellara del Belice and Canino, collected in August, and 3.96 for the drupes of the cv Tonda di Cagliari sampled in December. Oil content was extremely low in the olive fruits collected in August ranging between 1.45% and 5.73%, with the highest value -23.4%- recorded for the drupes of the cv Tonda di Cagliari collected in December. Consequently, a positive correlation (Pearson’s r = 0.87) was found between sampling date and oil yield, confirming what previously observed (e.g. Lavee and Wodner, 2004; Trentacoste et al., 2012).

**Table 1:** Sampling date, oil yield and Jaén index of the drupes of the 27 studied olive cultivars.

Cultivar	Sampling date	Oil yield (%)	Jaén index
Bidh el Hammam	08/02/2023	2.63	0
Grossa di Spagna	08/02/2023	1.45	0
Coratina (I)	08/02/2023	3.09	0
Tonda di Cagliari (I)	08/02/2023	5.73	0
Nocellara del Belice (I)	08/02/2023	3.67	0
Arbequina	08/02/2023	5.65	0
Canino	08/02/2023	3.05	0
Picholine Marocaine	10/03/2023	14.26	0.9
Pizzutella	10/03/2023	17.87	2.5
Picholine	10/10/2023	16.32	0.7
Toscanina	10/10/2023	16.61	1.7
Coratina (II)	10/18/2023	15.45	0.4
Grappolo	10/18/2023	14.56	1.9
Maurino	10/24/2023	14.82	1.4
Bouteillan	10/24/2023	17.09	1.6
Carolea	10/24/2023	20.10	1.4
Tonda di Cagliari (II)	11/06/2023	19.5	2.00
Nocellara del Belice (II)	11/06/2023	18.3	0.8
Moraiolo	11/06/2023	19.6	2.00
Minuta	11/14/2023	19.65	2.00
Semidana	11/21/2023	16.75	1.2
Tonda di Filogaso	11/21/2023	21.17	2.8
Koroneiki	11/27/2023	18.18	1.2
Frantoio	11/27/2023	20.57	1.6
Tonda di Strongoli	12/04/2023	17.49	1.5
Tonda di Cagliari (III)	12/07/2023	23.4	3.96
Nocellara del Belice (III)	12/07/2023	23.13	1.2
Ortolana	12/11/2023	12.99	1.8
Gentile di Larino	12/11/2023	19.54	2.00
Rastellina	12/11/2023	11.01	3.00
Corniolo	12/11/2023	20.37	3.6
Coratina (III)	12/18/2023	21.26	1.2
Colombina	12/18/2023	17.29	3.2

### 3.2 Comparison between the two extraction methods for evaluating FA composition

The identified fatty acids in all samples were typical of olive oil and consisted of palmitic (C16:0), palmitoleic (C16:1), heptadecanoic (C17:0), heptadecenoic (C17:1), stearic (C18:0), oleic (C18:1), linoleic (C18:2), linolenic (C18:3), arachidic (C20:0), eicosenoic (C20:1), behenic (C22:0) and lignoceric (C24:0) acid.

**Figure 1:**
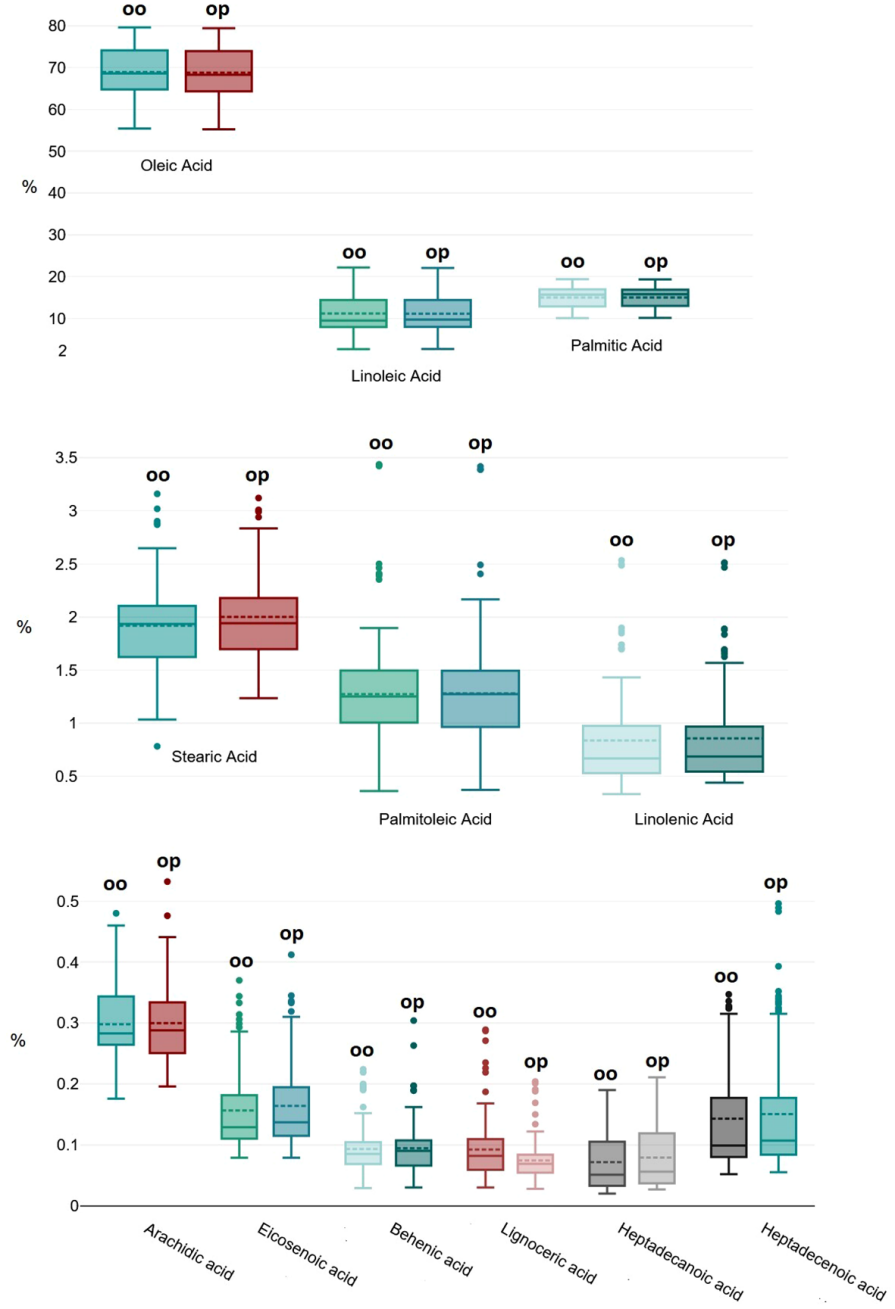
Overall distribution of the 12 recorded fatty acids extracted with the two different methods from the two matrices: olive oil (oo in the graphic) and olive paste (op).

MANOVA highlighted no significant difference (p=0.078) between the two extraction methods (from olive oil and olive paste) in determining the overall FA composition (**Figure 1**), thus proving the here proposed new method fully reliable and interchangeable with the official one. Interestingly, these results highlight an extreme uniformity in the FA composition of the drupes of a single plant, whereas only 10 g of drupes were found to be sufficiently and robustly representative for this analysis.

From the best of our knowledge, to date only a few authors carried out analysis of FA composition of oils from drupes/olive paste without firstly producing the oil by using an oil mill. Garcés and Mancha (1993) developed a procedure for carrying out digestion of fresh plant tissues, transmethylation of lipids and extraction of FAMEs in one step, by using 50-100 mg of olive fruits treated with a mixture of methanol, heptane, 2,2-dimethoxypropane and H_2_SO_4_ in specific proportions and a solvent like benzene, toluene or tetrahydrofuran, and heated at 80°C for 1-2 h in a water bath. The mixture, which forms a single phase during heating, extracts the lipids from the tissue and converts them into the corresponding FAMEs. After cooling, a separation of phases can be observed and FAMEs can be recovered in the upper layer. This protocol was adopted by De la Rosa et al. (2016) in the context of an olive breeding program, in order to evaluate quality oil parameters at early stages of the selection. Fruit samples were stored at -80°C and then lyophilized. After lyophilization, the stones were removed and the flesh was milled and stored at -20°C till analysis. FA composition of the oil was determined by simultaneous oil extraction and FA methylation as described by Garcés and Mancha (1993). Gómez-González et al. (2011) performed a traditional Soxhlet extraction for 24 h at 69°C on 15 g of milled drupes, using 80 ml of *n*-hexane. After extraction, they removed the hexane under vacuum using a rotary evaporator and the traces of water with a gentle N_2_ stream, before proceeding to the derivatization step and the chromatographic analysis. In order to study the olive ω-3 fatty acid desaturase gene family and identify the genes responsible for the linolenic acid content of olive oil, Hernández et al. (2016) performed analysis of lipids from olive mesocarp at different stages of development and ripening. After treatment of the olive mesocarp with isopropanol at 70°C for 30 min to inactivate endogenous lipases, lipids were extracted using a mixture of hexane:isopropanol, followed by a wash of the extract with aqueous sodium sulfate to remove non-lipid contaminants, as previously described by Hara and Radin (1978) for the extraction of lipids from rat and mouse brain. Lipids were subsequently separated by thin layer chromatography, subjected to transmethylation and analyzed by gas chromatography. Same protocol was adopted by Hernández et al. (2019) in a study aimed at the understanding of the impact of transcriptional regulation of stearoyl-acyl carrier protein desaturase genes on the content of unsaturated fatty acids in olive mesocarp under abiotic stresses.

Compared to the methods adopted by other authors, the protocol proposed in this study appears to be easier to apply, as it does not require many operational steps, nor the use of specific equipment, such as rotary evaporators, freeze dryers or TLC plates. Furthermore, only one solvent is used, the hexane, which presents less toxicity than other solvents, such as benzene or toluene. Eventually, this is the only experiment in which the results are compared to those obtained with an officially recognized method.

### 3.3 Comparison of FA composition in the 27 tested olive genotypes

**Figure 2:**
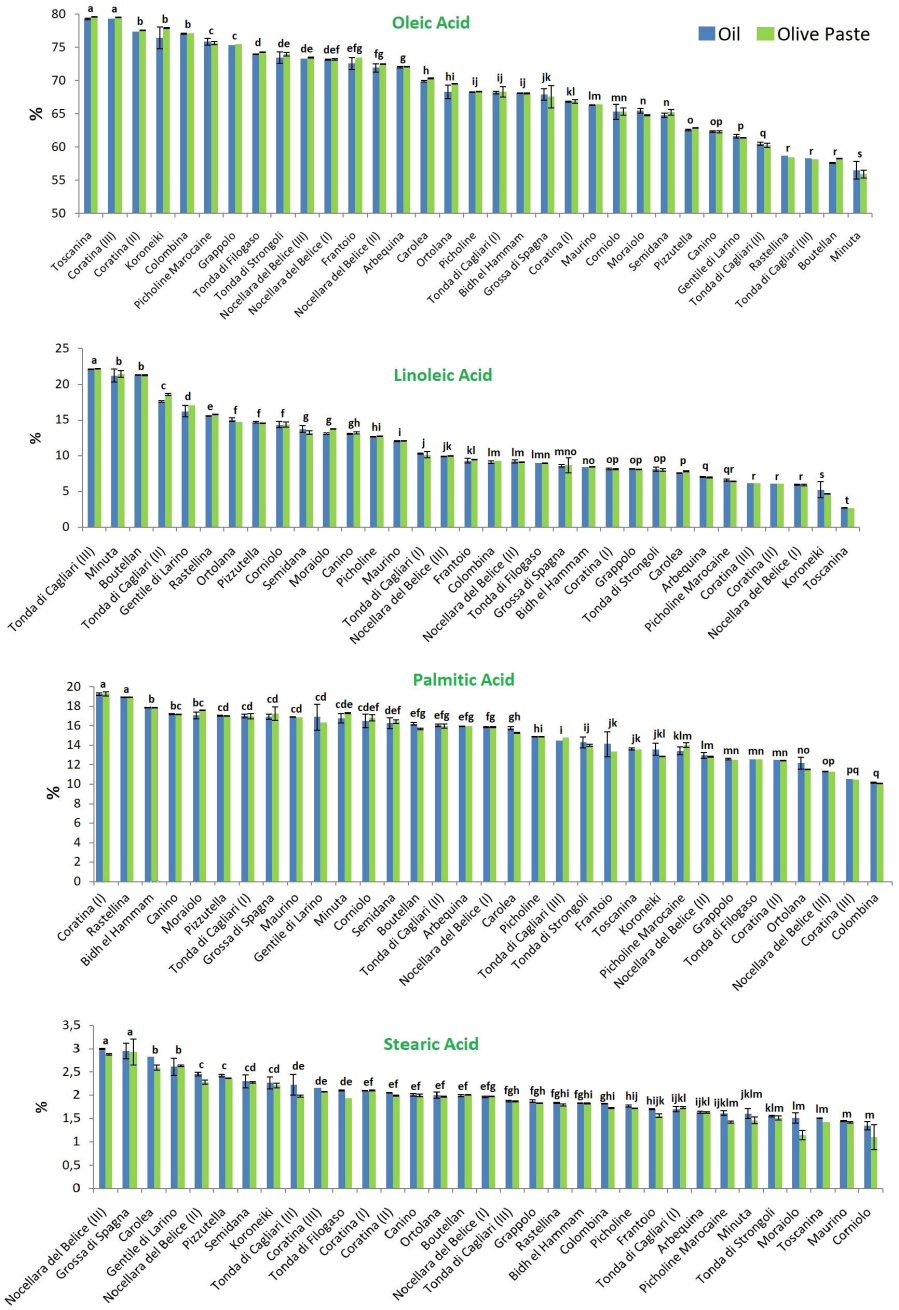
Analysis of the four most representative fatty acids in the 27 tested olive cultivars. Blue and green colors indicate the two extraction methods, from olive oil and olive paste, respectively. Different lowercase letters indicate statistical difference at p ≤ 0.05 level (HSD Tukey’s test).

The percentage composition of the four principal FAs (oleic, linoleic, palmitic and stearic acid, accounting on average for 97% of the total) in the 27 considered olive cultivars are reported in **Figure 2**. Again, no statistical differences between the extraction methods were found, but a great variability was encountered among the cultivars.

As expected, oleic acid was always the most abundant fatty acid. It was found at the mean concentration of 68.74 ± 6.68 g per 100 g of the total fatty acid composition when oil was extracted by mill, and 68.90 ± 6.43 g per 100 g when a solvent was used for the oil extraction. The October- and December-collected fruits of the cv Toscanina and Coratina (III), respectively, showed the statistically significant highest amounts of oleic acid, representing over 79% of the total. The absolute lowest percentage was found in the cv Minuta (~56%), statistically different from all the other cultivars.

**Figure 3:**
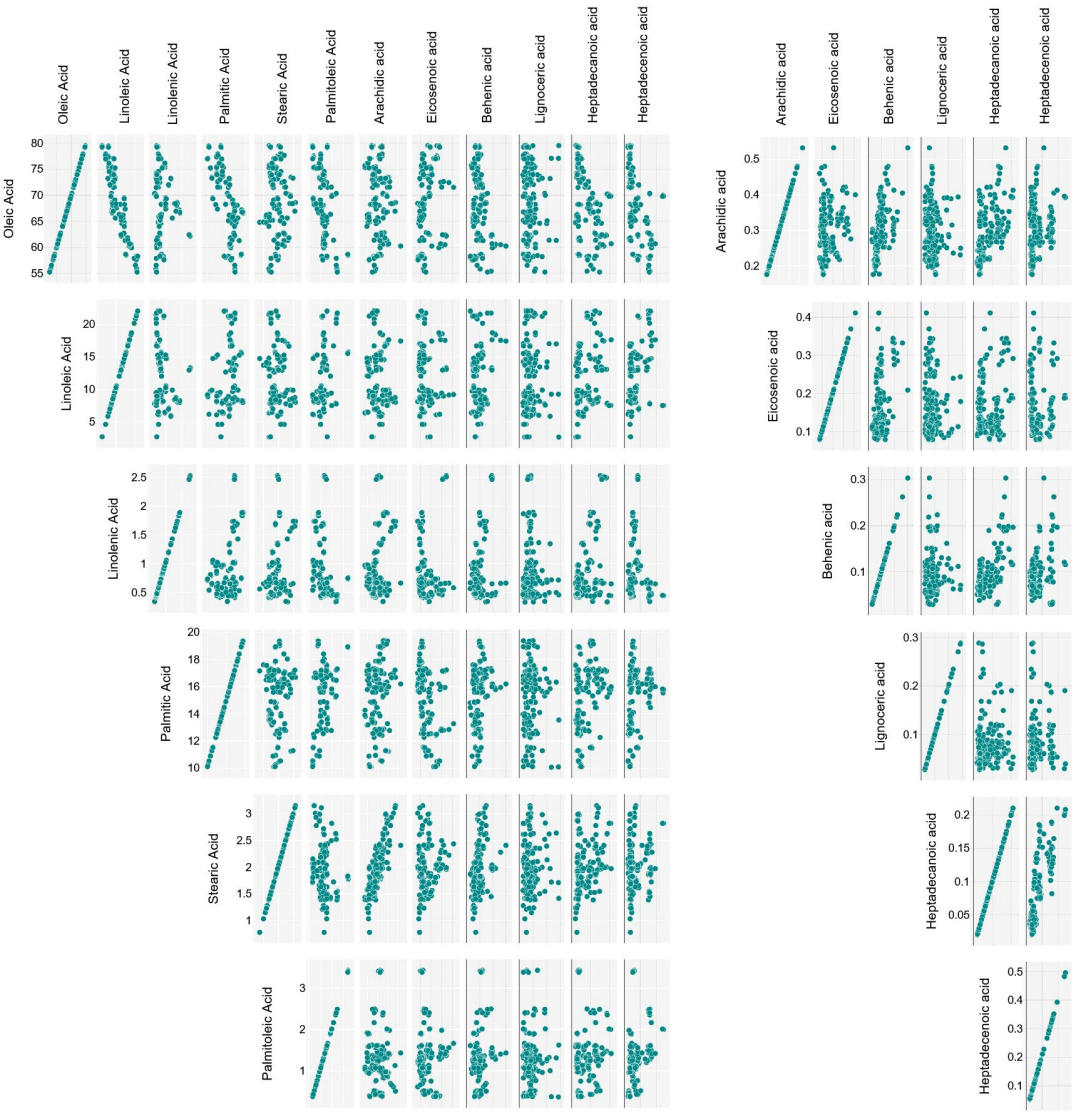
Correlation matrix of the 12 quantified FAs.

Linoleic acid content ranged between 2.68 ± 0.01 and 22.15 ± 0.05%, in Toscanina and Tonda di Cagliari (III), respectively, with mean concentrations of 11.16 g per 100 g. A strong negative correlation (Pearson’s r = - 0.9) was found between oleic and linoleic acid content (**Figure 3**), in accordance with literature (León et al., 2004; Rondanini et al., 2011; Sabetta et al., 2013; Ipek et al., 2015). This is a logical direct outcome of the inverse relationship of these two FAs, whereas linoleic acid is directly formed by desaturation of oleic acid, through the *FAD2-2* oleate desaturase gene (Salimonti et al., 2020). Nevertheless, no clear correlation between oleic and linoleic acid values was found in the oil of the cv Nocellara del Belice during the three ripening stages. Specifically, an overall stable concentration of oleic acid (73, 72 and 73%) on the three dates is accompanied by an almost twofold increase in linoleic acid (from 5.9 to 9.9%). Regarding this last aspect, the cultivars followed throughout the sampling period showed divergent behaviors. Given the pattern for the cv Nocellara del Belice (no relevant variations in the oleic acid content, increase of linoleic acid), the cv Coratina showed statistically significant drops in oleic acid content with ripening (and vice versa for linoleic acid), going from around 67% of the total FAs in August to 77% in October and 79% in December. A specular behavior was eventually assessed for the cv Tonda di Cagliari where oleic acid decreased from around 68%, to 60%, to 58%. Contrasting patterns have been described for oleic acid content during fruit ripening, showing a more or less firm decrease in the cvs Arauco, Arbequina, Barnea, Manzanilla Fina and Frantoio, while it has been described as constant in the cv Coratina from pit hardening to harvest and in the cv Arbosana and to increase in the cv Koroneiki (Rondanini et al., 2014; Bodoira et al., 2016; Polari et al., 2021). Similarly, in a three-year study in Calabria (Italy) Poiana and Mincione (2004) reported a net October to January oleic acid increase in the oil of the cv Cassanese, a slight increase for the cv Coratina -in accordance with this study-, Itrana, Sinopolese, Pendolino and Leccino, and a decrease for the cv Ottobratica and Nociara. More, a negative effect between mean daily temperature over 25°C and oleic acid proportion has been experienced in Argentina (García-Inza et al., 2018). On the contrary, the maximum summer temperatures were reported to increase oleic acid concentration, with an opposite trend during June and September-November period (Deiana et al., 2023). A certain role of temperature was suggested by Lombardo et al. (2008) evaluating the acid profile of 188 Italian cultivars at the same ripening stage in different years, with higher sums of growing degrees days leading to a decrease in oleic acid composition. Conversely, according to Tomé-Rodríguez et al. (2023) high temperatures together with reduced accumulated precipitation during fruit development induce a decrease of MUFAs and a consequent increase of saturated (SFAs) and polyunsaturated (PUFAs) FAs. In our study, the regulatory effect of the genotype, more than any other variable, appears particularly clear considering the notable difference in average temperatures during the sampling periods, passing from 29.7°C in August, to 18.9°C in October, to 11.6°C in December.

Palmitic acid was the most dominant SFA in all samples investigated, present at a mean concentration of 15.01 ± 2.42% *vs* 15.02 ± 2.41% in mill *vs* solvent extraction. Its content varied between 10.10 ± 0.02% and 19.28 ± 0.21%. The lowest amount of palmitic acid was found in the cv Colombina, while the statistically highest values were found in Coratina (I) and Rastellina.

Stearic acid was found at the mean concentration of 1.96 ± 0.44%, with the uppermost extreme of 3% recorded for both the cvs Nocellara del Belice (III) and Grossa di Spagna, while the cvs Moraiolo, Toscanina, Maurino and Corniolo showed the statistically significant lowest values (average of 1.46 ± 0.11, 1.43 ± 0.01, 1.32 ± 0.01 and 1.22 ± 0.09% respectively).

Regarding the “minor” fatty acids, an interesting and original finding was that linolenic acid in the oils of the drupes collected in August was always found to be above the limit value of 1% for the classification of olive oils (**Table 2**), falling well below the threshold on other dates. The early fruiting cultivar Tonda di Cagliari presented the lowest value, just above the limit, of 1.02%; this could somehow suggest an influence of drupe development on the biosynthesis of this fatty acid. However, it cannot be ruled out that the high summer temperatures may also have some effect. In this vein, two fatty acid desaturase genes (*FAD7-1* and *FAD7-2*) have been suggested as responsible for linolenic acid content in olive mesocarp, with high *FAD7-1* expression levels recorded in young drupes of the cv Picual and Arbequina (Hernández et al., 2016). The higher oil yield justifies the higher total linolenic acid content found as the drupe ripens. Nevertheless, FAD7 has been proposed as negatively affected by high temperatures in a work with olive callus cultures (Hernández et al., 2008). Further investigations are needed to clarify this little studied aspect.

**Table 2:** Percentage of linolenic acid in the oil from the drupes collected in August.

Cultivar	Linolenic acid (%)
Coratina (I)	1.87 ± 0.02
Nocellara del Belice (I)	1.35 ± 0.01
Arbequina	1.20 ± 0.01
Bidh el Hammam	1.69 ± 0.01
Canino	2.50 ± 0.02
Grossa di Spagna	1.63 ± 0.12
Tonda di Cagliari (I)	1.02 ± 0.01

All the other FAs resulted to be within the limits defined by law, with the levels of heptadecenoic acid in the cvs Carolea (0.49%), Pizzutella (0.32%) and Tonda di Cagliari II and III (0.34 and 0.33, respectively) confirming the need to raise the limit to 0.6% in the European Union, previously set at 0.3% like currently in other international standards (UC Davis Olive Center, 2018).

Considering the 12 fatty acids, apart from the above described oleic-linoleic acid relation, statistically significant positive correlations were also found between stearic and arachidic acid (r = 0.83), heptadecanoic and heptadecenoic acid (r = 0.82), and palmitic and palmitoleic acid (r = 0.56), and negative ones between oleic acid and palmitic (r = -0.71), palmitoleic (r = -0.56), heptadecenoic (r = -0.56) and heptadecanoic (r = -0.49) acid (**Figure 3**).

The palmitic-palmitoleic acid and stearic-arachidic acid positive correlations have been previously reported (e.g. Stefanoudaki et al., 1999; León et al., 2004; Revelou et al., 2021), like the negative ones between oleic-palmitoleic acid and oleic-palmitic acid (Rondanini et al., 2011; Revelou et al., 2021). On the other hand, the relationships with and between heptadecenoic and heptadecanoic acids appear to have not been yet investigated. Although described by other authors (Kritioti et al., 2018; Revelou et al., 2021), only a weak negative correlation was found between oleic and stearic acid.

**Figure 4:**
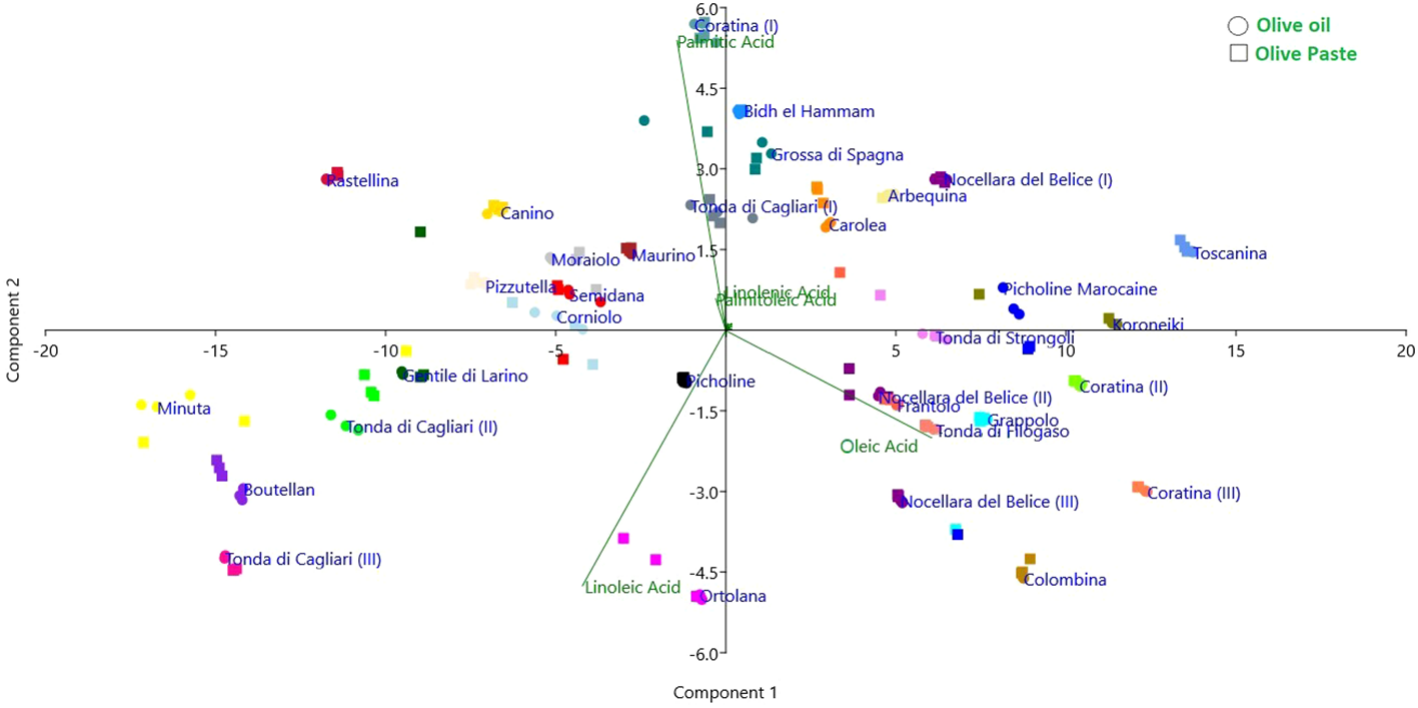
Principal component analysis showing cultivar distribution according to FA profile and extraction method.

Eventually, principal component analysis (PCA) allowed to visualize the spatial clustering of the cultivars taking into account the extraction method and the total composition of the fatty acids (**Figure 4**). This analysis also confirmed the general comparability of the two extraction techniques, the net separation of the three ripening stages of the cvs Coratina and Tonda di Cagliari, and a substantial separation of the high oleic cultivars (including Colombina, Nocellara del Belice, Tonda di Filogaso, Grappolo and Coratina at the two most advanced stages of maturation considered) from the high linoleic (like Ortolana, Tonda di Cagliari II and III, Boutellan) and palmitic (Coratina I, Bidh el Hammam, Grossa di Spagna) ones. If these groups confirm the negative correlations previously described, an exception is represented by the cv Ortolana showing good levels of oleic acid (69%) and high values of linoleic acid (14%).

## 4 Conclusions

The method here described for oil extraction through a volatile solvent resulted to be a reliable (no statistical difference) and rapid option to olive milling for the analysis of FA composition. The great advantage of this technique is that with a single sampling of only a few olives it is possible to determine oil yield and FA composition, so as to be able to set the most suitable harvest calendars. This method can prove useful, especially in years of low production or in the earliest stages of drupe development when the oil yield is still low, also in studying the trend of FA composition which still shows many dark sides. In this regard, the preliminary results here presented suggest that there is not a common trend of the individual FAs during the olive ripening process. The trend could be rather influenced by varietal factors, but further studies on a greater number of cultivars are necessary.

## Data Availability

The raw data supporting the conclusions of this article will be made available by the authors, without undue reservation.
